# Effectiveness of Teacher-Led Nutritional Lessons in Altering Dietary Habits and Nutritional Status in Preschool Children: Adoption of a NASA Mission X-Based Program

**DOI:** 10.3390/nu11071590

**Published:** 2019-07-13

**Authors:** Jieun Kim, Gilsook Kim, Jinah Park, Youfa Wang, Hyunjung Lim

**Affiliations:** 1 Department of Medical Nutrition, Graduate School of East-West Medical Science, Kyung Hee University, Yongin 17104, Korea; 2 Department of Early Childhood Education, Sahmyook University, Seoul 01795, Korea; 3 Korea Institute, of Child Care and Education, Seoul 06750, Korea; 4 Systems-oriented Global Childhood Obesity Intervention Program, Department of Epidemiology and Environmental Health, School of Public Health and Health Professions, University at Buffalo, State University of New York, New York, NY 14214-8001, USA

**Keywords:** childhood obesity, dietary behavior, early childhood, nutrition education, prevention

## Abstract

The preschool years are a sensitive period for the development of food preferences that will affect physical growth and life-long health. The promotion of healthy eating and nutritional status was achieved by adapting the US National Aeronautics and Space Administration (NASA) Mission X (MX) Program among young children in South Korea. The intervention program was delivered by nutritional experts and class teachers over 10 weeks. Children from 37 school classes (*n* = 534) from 7 daycares and kindergartens were randomized into a control group (CG, *n* = 280) and an intervention group (IG, *n* = 254). Parents were surveyed for their children’s characteristics and nutrition quotient (NQ) at baseline and at the 10-week follow-up. At baseline, 18.8% (boys: 18.9%; girls: 18.8%) of the subjects were overweight or obese (body mass index ≥ 85th percentile). After the intervention, the mean differences in various anthropometric measures did not differ significantly between the groups in a linear regression model adjusted for age, sex, and type of school. The NQ grades were significantly higher in the IG than the CG after the intervention (*p* = 0.000). In summary, the 10-week South Korean MX program improved the eating behaviors and nutrition status of young children. A further multisector prevention program is needed to prevent childhood obesity in young children.

## 1. Introduction

Early childhood is the developmental stage in which growth occurs and diet- and health-related behaviors are established [1]. Previous research has demonstrated that the formation of even one unhealthy dietary behavior, such as higher consumption of high-energy dense snack consumption, over- or undernutrition, and food preferences, in early childhood affects health for a lifetime [2,3]. Nutrient-poor food choices and excessive fat development in early childhood are associated with a higher risk of long-term non-communicable diseases, including obesity [4,5].

The National Children’s Health Examination in South Korea indicated that 42.5% of young children with obesity in this population were picky eaters, and 32% watched TV for ≥2 h/day based on mandatory health checkup data [6]. Moreover, during the last 10 years, fat consumption and sugar-sweetened beverage intake have been increasing, while calcium, fruit, and vegetable consumption have been decreasing in all age groups, including in children 3 to 5 years old [7].

Globally, the rise of the importance of the early childhood education setting (ECES) has been reported [8]. Early childhood education and care have a major role for improving child development or also as an alternative to family care following the development of the economic status and growing population of working mothers of preschool children [7]. During the preschool period, healthy dietary habits and food preferences are learned and formed at home, in early childcare centers, and in kindergarten. Children obtain more than 70% of their daily nutrition intake in childcare settings [9]. In South Korea, the Center for Child-Care Foodservice Management was conducted to improve the variety level of diet provided in child-care centers and kindergartens based on the Korean dietary reference intake (KDRI, 2015) [10]. However, different levels of nutritional status and unhealthy eating habits, such as meal regularity and breakfast skipping and higher consumption of energy-dense/nutrient poor food, are found in young preschool aged children attending kindergarten or daycare centers [11,12].

Several nutrition education programs have reduced unhealthy dietary behaviors in young children in Korea [12,13,14]. Improvements in the nutrition knowledge score [12], nutritional status balance, and regular diet with a higher frequency of consumption of white milk [13] were shown after the nutritional education. Furthermore, vegetables & fruits (V/F) consumption and healthy eating behaviors were higher in the education group than in the control group. Another previous study reported an improvement of fruit and vegetable intakes as well as preschool children’s emotional and behavioral outcomes after nutrition education [14]. Therefore, school-based nutritional education needs to be considered in ECES.

A recent review demonstrated that expert-led multilevel interventions with parental and teacher engagement successfully altered the diet- and health-related behaviors and anthropometric outcomes of preschool-aged children [15]. Nevertheless, no study has used a multicomponent approach with expert nutrition team-based training sessions in a large sample of South Korean preschool children. The National Aeronautics and Space Administration (NASA) established the Mission X: Train Like an Astronaut (MX) program to promote diet and fitness in children by motivating them with astronauts’ real training program [16]. We previously found that a 6-week MX program improved the dietary balance and nutritional knowledge of young preschool children [17].

We aimed to improve the dietary behaviors and nutritional status of preschool children through a 10-week nutrition-themed MX program.

## 2. Materials and Methods

### 2.1. Setting and Subjects

The setting for this study included middle-socioeconomic-class daycares and kindergartens. The Korea Institute of Child Care and Education (KICCE) recruited through daycares and kindergartens by means of a screening process and interviewing the class teachers and directors. Seven daycares and kindergartens (1) located in the Gyeonggi area; (2) with middle-income households; and (3) with healthy 4- to 5-year-old preschoolers were included. The KICCE recruited 37 classes (*n* = 679) from the 7 daycares and kindergartens by random sampling for the present randomized controlled study. Three to seven classes from each school were randomly divided into the intervention group (IG = 339) or the control group (CG = 340). Two groups (IG and CG) of the participants were from each of the same daycare and kindergarten, by being assigned random numbers, into two groups. Among the children, those (1) who did not want their height and weight measured, (2) or were absent or moves to another area from their school were excluded. All families that completed a consent form (both parent and child) and the questionnaires were enrolled. This study was approved by the institutional review board of The Korea Institute of Child Care and Education (KICCEIRB-2016-03).

### 2.2. Nutrition-Themed Intervention and Key Concepts

A 10-week nutrition intervention was performed by a team of nutrition professionals with the cooperation of 14 class teachers at the 7 daycares and kindergartens from May to August 2016. The nutrition professional team consisted of two registered dietitians and six dietitians from the Research Institute of Medical Nutrition at Kyung Hee University. Each part of the intervention was performed by the nutrition professional, and class teachers taught using the provided lesson plans and materials. All the nutrition sessions were implemented in the intervention groups of the seven daycares and kindergartens during the same week.

The 10-week nutrition-themed Mission X program was developed by a committee of the KICCE and nutrition professionals, based on four components of the original National Aeronautics and Space Administration (NASA) Mission X program [16] and the previously modified Korean version of the MX program [17] (Supplementary Table S1). The second version of the nutrition curriculum consisted of four nutrition-themed components from the original U.S. Mission X program: 1. Energy of an astronaut, 2. Hydration station, 3. Living bones, strong bones, and 4. Reduced gravity, low-fat [16]. The theme and goal of each session were maintained, while the activities and materials of the nutrition sessions were modified.

The four nutrition-themed sessions were delivered by a nutritional expert team and the class teachers according to the concept (i.e., science, game activity) of each class. The energy of an astronaut aimed to teach students about balanced meals and specific nutritional needs by asking them to categorize and pair different food items based on the five food groups. Previously used educational materials, such as movie clips, food balance wheels, and food models, were used for this first session of the program. The second session, hydration station, included think and tell, comparison, game-playing, and observation methods to highlight the importance of water for health, the signs of dehydration, and the relationships between the body systems/organs and hydration. The third and fourth sessions aimed to teach students to recognize calcium-rich foods and formulate healthy food choices. For the living bones, strong bones session, educational movie clips comparing different bone shapes and structures were played for the class. Lastly, the reduced gravity, low-fat session employed a fat vest and a healthy vs. unhealthy snack board to allow students to discover the fat and sugar contents of unhealthy foods and snacks. All the key concepts of the nutrition sessions were equally offered to the control group after the 10-week follow-up.

### 2.3. Nutrition Session Training

The curriculum of the four nutrition-themed sessions were delivered to the class teachers of the seven daycares and kindergartens and the nutrition professional team by a senior nutrition professional in a total of three 30-min training sessions. During the training sessions, the class teachers were provided with lesson plans and materials for teacher-led nutrition-themed activities and were educated in the key concepts of the MX program and the themed nutrition sessions. The training schedule was organized by the KICCE with consideration of the schedule of each daycare and kindergarten and the possibility of teacher participation.

For the energy of an astronaut session, the roles of the five food groups in a balanced diet were represented in the classroom through a game of pairing food items and a lunchtime food mission based on the five food groups. The hydration station was modified based on seasonal events and the schedule of the ECES. The key concept was delivered through practical nutritional messages in the class and through outdoor activities to promote drinking eight cups of water per day. For the ninth and tenth weeks of the intervention period, the teachers organized their class time and various themed activities around the nutritional messages.

After each training session, the nutrition professional team organized a visit schedule for the four nutrition-themed sessions with the class teachers. During the 10-week intervention, the nutrition professional team visited the seven daycares and kindergartens to perform the four nutrition sessions, in accordance with the study protocol.

### 2.4. General Characteristics

Before the MX program, the teachers distributed consent forms and questionnaires to the preschoolers and their parents. At baseline, the parents were surveyed about their children’s general characteristics, including birth-related characteristics (birth length, birth weight, and gestational age) and diet-related characteristics (breastfeeding, picky eating, and supplement usage).

### 2.5. Anthropometric Measurements

In accordance with the study protocol, all preschoolers’ anthropometric data were measured by trained research staff. Height was measured to the nearest tenth of a centimeter (cm) with a mobile (Body COM, HM 002, Seoul, South Korea), and weight was measured to the nearest tenth of a kilogram (kg) on a portable digital scale (CAS, PB-500, Yangju, Gyeonggi-do, South Korea) [18].

Body mass index (BMI) was calculated with the standard equation (body weight [kg]/height [m]^2^). We used the sex-age-specific BMI percentiles from the Korean CDC Growth Chart (KCDC, 2017) [19] to assess each child’s weight status: Those in the 85th percentile and above were considered at risk of being overweight, while those below the 10th percentile were considered at risk of being underweight. The rest were considered to have a normal BMI.

### 2.6. Assessment of Nutrition Quotient (NQ)

The children’s dietary behavior, as self-reported by their parents, was assessed with the nutrition quotient (NQ) [20,21]. The NQ was developed by the Korean Nutrition Society in 2012, and the construct was validated in a previous study [21]. The NQ was newly used as a comprehensive system to evaluate children’s dietary quality, eating attitudes, and behaviors. The NQ questionnaire consists of 19 items grouped into five factors: Balance (mixed grains, milk, legumes, eggs, and fruit), diversity (diverse side dishes, kimchi, and vegetables), moderation (fast food, instant noodles, late-night snacks, street food, and sweets), regularity (meal regularity, eating breakfast, and watching TV/playing computer games), and practice (checking nutrition labels, chewing well, and washing hands before meals). The scores of all five factors were summed to yield the total NQ score, ranging from 0 to 100. Five different grades (highest: 80.9–100, high: 73.8–80.8, medium: 56.5–73.7, low: 47.6–56.4, lowest: 0–47.5) were assigned according to total NQ score. We represented that from high to highest for ‘high’, and low to lowest for ‘low’ of the NQ score.

### 2.7. Outcomes

NQ score and grade were the primary outcomes and the secondary outcome was anthropometric measurements (height, weight, and BMI) of preschool children.

### 2.8. Statistical Analysis

We compared the differences in dietary behaviors between the allocated study groups (IG vs. CG) using the *t*-test and Chi-square test. Paired *t*-tests were performed to determine the statistical significance of mean differences in variables between the pre- and post-tests. The mixed effects linear regression model was used to assess changes in anthropometric variables and NQ between the groups after adjustment for age, sex, and type of school (daycare or kindergarten).

All statistical analyses were performed in SPSS version 23 for Windows (SPSS Inc., Chicago, IL, USA). The results are expressed as means and standard deviations for continuous variables and numbers and percentages for discrete variables. Statistical significance was defined as *p* < 0.05.

## 3. Results

**Figure 1:**
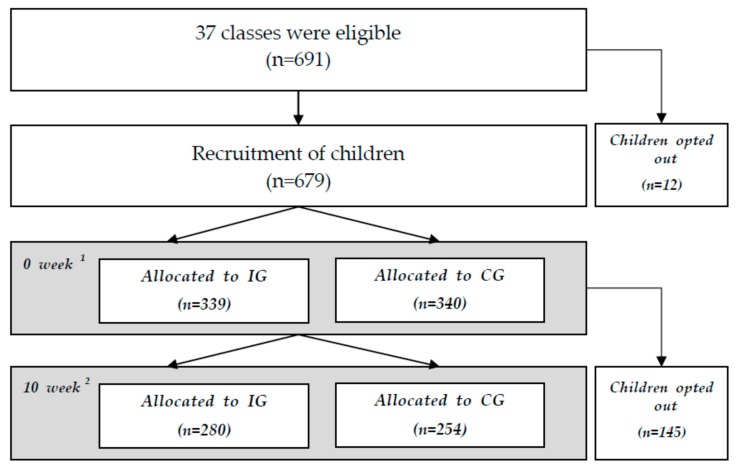
Intervention procedures following as the study protocol. ^1^ 0 week (baseline): anthropometric measurement (n = 679), self-reported demographic and NQ questionnaires (n = 534), ^2^ 10 week (follow-up): anthropometric measurement (n = 612), self-reported demographic and NQ questionnaires (n = 534). This study was approved by the institutional review board of The Korea Institute of Child Care and Education (KICCEIRB-2016-03).

Figure 1 displays the intervention procedures of the study in accordance with the study protocol. Overall, 691 preschool-aged children were enrolled from 37 classes in seven childcare settings in Korea. At baseline, 12 preschool children opted out due to illness or quitting the daycare or kindergarten. In total, 679 children participated in the MX program, and 534 (78.6%) completed the intervention, and pre-post assessments and were included in the analyses (Figure 1).

### 3.1. Demographic Characteristics of the Study Subjects

The demographic characteristics of the children are summarized in Table 1. The majority of children attended a daycare center (54.6%). In total, 52.9% of the children were in 4-year-old classes, and 56.4% were boys. Approximately 6.3% of the children were categorized as underweight, while 11.0% were overweight or obese. Among the subjects, 86.9% were breastfed, half were picky eaters, and 49.0% took supplements regularly. No significant differences in birth- and diet-related characteristics (birth length, birth weight, gestational age, breastfeeding, picky eating, and supplement usage) were observed between the intervention and control groups (Table 1).

### 3.2. Pre-to-Post-Intervention Changes in Anthropometric Measurements

Pre-to-post-intervention changes in BMI are shown in Table 2. The mean BMI (kg/m^2^) at baseline was 16.2 ± 2.4 for the boys and 15.9 ± 1.5 for the girls (data not shown). Significant changes in anthropometric variables were observed in both groups after the intervention (*p* < 0.05). However, none of the mean differences differed significantly between the groups in the mixed effects linear regression model adjusted for age, sex, and type of school.

### 3.3. Pre-to-Post-Intervention Changes in Children’s Dietary Behavior Based on NQ Score

Pre- and post-intervention measures of NQ (balance, diversity, moderation, regularity, and practice) by study group are shown in Table 3. The total NQ score of the IG increased significantly (from 64.1 to 66.0, *p* < 0.05) after the intervention program. A change in fruit consumption of the balance factor was significantly greater in the IG than in the CG (2.0 vs. −1.5, *p* < 0.05). The moderation factor score, which evaluates the intake of energy-dense and high-sodium foods (fast food and instant noodles), was significantly higher in the IG than in the CG (0.5 vs. −1.2, *p* < 0.05). Changes in meal regularity of the regularity factor was significantly greater in the IG than in the CG (3.4 vs. 1.2, *p* < 0.05). Among the practice factors, hand-washing received significantly higher scores in the IG than in the CG (4.6 vs. −0.7, *p* = 0.000) (Table 3).

### 3.4. Pre-to-Post-Intervention Changes in Children’s Nutritional Status Based on NQ Grade

**Figure 2:**
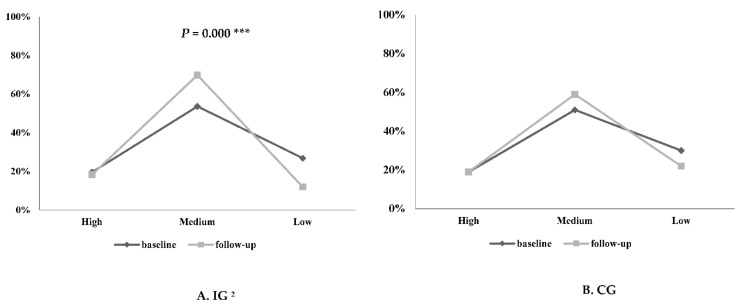
Changes in the nutrition quotient (NQ) grade of the preschoolers participating in the Mission X program according to their group. ^2^ IG: Intervention group, CG: control group. ***: *p* < 0.001 by McNemar test.

In both groups, all preschool children were divided into low to high grades of the NQ according to their nutritional status (Figure 2). The NQ grades differed significantly between the IG and the CG only after the intervention (*p* = 0.000). At baseline, 26.8% of participants were in the low, 53.6% in the medium, and the rest of the participants were in the high grade of the NQ in the IG. After the intervention, only 11.9% of the low and 69.8% of the medium participants were observed in the IG.

## 4. Discussion

The present study evaluated the effectiveness of a 10-week nutrition-themed intervention to improve the eating habits and nutritional status of preschool-aged children attending daycare centers or kindergartens in South Korea. The intervention targeted the daycare and kindergarten curriculum and provided teacher support. Certain healthful dietary behaviors, such as increasing fruit consumption and reducing energy-dense food and snack consumption, were improved in the IG compared to the CG. However, none of the mean differences in anthropometric variables (BMI, BMI-z, BMI-SDS) were found to differ between the IG and the CG in the regression model. These findings suggest that a school-based intervention involving teacher training, educational materials, and activities improved the nutritional status of preschool-aged children and promoted food-related behaviors that prevent childhood obesity.

Globally, 42 million children under the age of 5 years are overweight or obese [22]. A recent population-based longitudinal childhood obesity study described the persistence of excess weight gained between the ages of 4 and 6 years [23]. A higher BMI caused by excessive fat accumulation in early childhood hastens the onset of obesity and non-communicable diseases (NCDs) later in life. Previous systematic reviews have suggested that school-based childhood obesity prevention studies can effectively reduce the proportion of overweight children [24,25]. Another study demonstrated that the mean BMI z-score was non-significantly lower in the intervention arm after an obesity prevention program delivered through a school [26]. In line with this finding, none of the mean differences in anthropometric measures differed between the groups in the present study; however, the BMI status of each group was significantly lower after the program. This might be explained by natural growth and seasonal changes that contribute to the development and weight status of young children. Additionally, within each ECES, the children and class teachers of the two groups mixed frequently for school activities and lunch. This may have motivated children in the CG to improve their weight status. Both IG and CG were in a normal weight status before and after the 10-week intervention study. It showed the harmless and valuable sides of the MX-program to promote physical growth and development in preschool children.

According to the World Health Organization (WHO, 2017), a healthful diet and lifestyle are key factors within a sustainable supportive environment and community [27]. It is well known that young children’s unhealthy eating habits and food choice are connected to long-term health consequences on mortality and morbidity in adulthood [28]. Howerton and colleagues analyzed seven previous studies and found that nutrition education effectively reduced unhealthy dietary behaviors in children [29]. Consistent with this, reduced unhealthy dietary behaviors, such as high intakes of discretionary foods, fast foods, and instant noodles, and increased V/F and dairy food consumption, [12,13,14] and nutrition knowledge score and nutritional status with balanced and regular diet were reported after the educational intervention in South Korea. Likewise, in preschool-aged children from our study, healthful dietary behaviors were improved in the IG, while unhealthful eating habits were reduced. Previous studies were implemented in a childcare center with only 6-year-old children [12] or focusing on only single content-V/F consumption [13,14]. In the present study, a large number of 4 to 5 preschool children participated in a nutritional theme-based MX-program for 10 weeks to promote children’s nutritional status and eating behaviors. Nutritional improvement in young children’s eating habits, dietary intake, and V/F intake, and decreased intake of total fat and saturated fat, fewer sweeten beverages, and poor energy dense and nutrient foods were effective dietary outcomes in the preschool setting intervention to prevent diet-related disease, including childhood obesity [15]. Therefore, our study showed the effectiveness of the nutritional intervention focusing on food and dietary factors.

In 2014, a 6-week MX intervention program was adapted from the original NASA Mission X (MX) program to prevent obesity among 5-year-old preschool children in South Korea [17]. Desirable improvements in dietary balance, healthful food intake, and nutritional knowledge were found in young children in that study. Consistent with this previous research, we found that the total NQ score (the diversity and practice factors in particular) increased only in the IG after the intervention. In addition, 15% of the children in the IG with a low score improved to the medium score, while no change in score was observed in the CG. Meanwhile, increased consumption of fast food and late-night snacks was found in the CG. These results indicated the feasibility of preventing childhood obesity by reducing unhealthy eating behaviors in young children.

Overconsumption of energy-dense and nutrient-poor foods is associated with increased risks of obesity, cardiovascular disease, and metabolic abnormalities [30]. Although the childcare setting is a unique place in which young children establish diet- and health-related behaviors, the home setting remains the predominant environment for discretionary food intake to prevent childhood obesity [31]. Therefore, caregivers at home and class teachers in ECES should share the responsibility of fostering healthful dietary habits in preschool-aged children.

The US MX program addresses healthful eating and fitness through the theme of human space exploration [16]. The content of this program is flexible, with various MX activities and web-based materials that can be modified and implemented in different settings at no cost. ECES could be an opportune setting for the modification of diet- and health-related behaviors in a large number of preschool children to support their healthy growth and development [3]. Previous systematic reviews have found that interventions in ECES significantly improved young children’s fruit and vegetable intake [32,33] and reduced their total and saturated fat intake. In particular, multicomponent interventions addressing both dietary and physical activity behaviors led to significant changes in children’s weight status [29]. However, no differences in anthropometric outcomes have been reported compared with strategies delivered by professionals. Even fewer positive diet-related outcomes have been found in childcare educator-led studies [30]. Another previous study, educational training and technical assistance of multi-component intervention, was supportive of implementation by organizing obesity-related food availability, classroom activities, and practices [34]. Additionally, teachers’ knowledge and self-efficacy affect the improvement of the health and nutritional status of preschool children by facilitating a nutrition and PA promoting environment in their classroom. The Toy Box-study reported that health promotion efforts with a multifactorial evidence-based approach and obesity prevention strategies by employing behavioral models found that increased the total diet quality and dietary equilibrium in the intervention group of European preschool children [35,36,37].

For the educational component, staff group education and training sessions with educational materials and activities are mostly used in childcare settings [38]. The present nutrition-themed MX program for young children was delivered by a nutrition professional team together with class teachers for the 10-week intervention period. Curricula promoting healthful behaviors, such as eating balanced meals, understanding the importance of water, consuming calcium-rich foods, and eating healthy foods and snacks, were designed to help children make good lifestyle choices related to food and nutrition. These nutrition-themed sessions may have helped the children to choose foods based on the role-model of an astronaut with a real training course. Furthermore, these themes allowed teachers to reinforce the nutrition concepts in their own classes. During the 10-week intervention with four different food- and nutrition-related strategies, preschool children played games (pairing food items based on the five food groups, hydrating an astronaut), observed examples (simulated urine samples, different bone shapes and structures), and discovered the fat and sugar contents of food items. This complementary delivery system effectively improved diet-related behaviors in young children.

A major strength of this study is that it is the first to promote healthful dietary behaviors in South Korean preschool-aged children by adapting four nutrition-related components of the NASA MX program with the cooperation of ECES class teachers. This study demonstrated that obesity-related constructs in a nutrition-themed approach could be implemented by nutrition staff and trained teachers using educational materials, science-based experiments, and games/activities. Lastly, the same nutrition sessions, activities, and materials were provided to the CG by the nutrition team staff after the 10-week intervention.

Nevertheless, there were a few limitations to the present study. The participating preschool-aged children were stratified sampled from middle-income households in only one region within the Gyeonggi area of Korea. This might limit the generalizability of our findings to other childcare settings, regions, or socioeconomic classes of other countries. However, we recruited two types of preschools, which is representative in Korea. Although some of the findings were favorable, children’s dietary behaviors were assessed based on their parents’ self-report of NQ to improve the rate of response. Further tools with ease of access are needed to evaluate the nutritional status of young children in school settings.

## 5. Conclusions

In conclusion, the effectiveness of a 10-week South Korean NASA MX-based program was evaluated by using NQ, 3% of the total NQ score, and 20% of the medium grade nutritional status of young children aged 4 to 5 years in the IG after the intervention program. This finding indicated that the effectiveness of a teacher-led NASA MX-based program, which is focused on learning the science behind nutrition and physical activities. This easily acceptable program, such as web interactive education materials focusing on fitness and health, can be an effective means of improving nutrition and health-related behaviors in the school or community setting. Sustainable and multisector prevention programs need to be implemented to promote early childhood development, healthy eating behavior, and an active lifestyle in real-life settings.

## Figures and Tables

**Table 1:** Demographic characteristics of the intervention group (IG) and control group (CG).

Variables	Total (*n* = 679)	Intervention Group (*n* = 339)	Control Group (*n* = 340)
Type of school (*n*, %) ^1^			
Daycare center	371 (54.6) ^1^	155 (45.7)	216 (63.5)
Kindergarten	308 (45.4)	184 (54.3)	124 (36.5)
Age (*n*, %)			
4 years	359 (52.9)	166 (49.0)	193 (56.8)
5 years	320 (47.1)	173 (51.0)	147 (43.2)
Sex (*n*, %)			
Boy	383 (56.4)	182 (53.7)	201 (59.1)
Girl	296 (43.6)	157 (46.3)	139 (40.9)
Body mass index (*n*, %) ^2^			
BMI < 10th%ile	41 (6.3)	22 (6.8)	19 (5.9)
10th %ile ≤ BMI < 85th%ile	535 (82.7)	269 (83.0)	266 (82.3)
85th%ile ≤ BMI	71 (11.0)	33 (10.2)	38 (11.8)
Birth-related (mean ± SD) ^3^			
Birth length (cm)	50.7 ± 4.4	50.8 ± 4.1	50.6 ± 4.7
Birth weight (kg)	3.2 ± 0.9	3.2 ± 1.2	3.3 ± 0.4
Gestational age (weeks)	39.2 ± 2.3	39.2 ± 2.2	39.1 ± 2.5
Diet-related (*n*, %)			
Breastfeeding	464 (86.9)	244 (88.1)	219 (85.2)
Picky eating	265 (49.6)	130 (46.9)	134 (53.2)
Supplement usage	317 (49.0)	166 (59.9)	151 (59.9)

^1^ Values are presented as n (%) or mean ± SD. ^2^ Anthropometric measurements at baseline (n = 647). ^3^ Self-reported demographic questionnaires at baseline (n = 534). No significant difference between groups was found by the Chi-square test for categorical variables and Student’s *t*-test for continuous variable.

**Table 2:** Changes in anthropometric measurement of the preschoolers after participating in the Mission X program.

Variables	Intervention Group (n = 280)			Control Group (n = 254)			
	Baseline	Follow-Up	∆ Change [95%CI]	Baseline	Follow-Up	∆ Change [95%CI]	*p* ^†^
Height (cm)	110.9 ± 5.7 ^1^	112.6 ± 5.8 ***	1.7 [−0.2, 0.1]	110.3 ± 6.0	112.0 ± 6.0 ***	1.6 [−0.2, 0.1]	0.907
Weight (kg)	19.7 ± 2.8	19.9 ± 3.0 ***	0.2 [−0.2, 0.0]	19.6 ± 3.1	19.9 ± 3.4 *	0.3 [−0.2, 0.0]	0.215
BMI (kg/m^2^) ^2^	16.0 ± 1.4	15.7 ± 1.5 ***	−0.3 [−0.1, 0.0]	16.1 ± 1.5	15.8 ± 1.6 ***	−0.3 [−0.1, 0.0]	0.268
BMI-Z	0.03 ± 1.03	−0.21 ± 1.10 ***	−0.23 [−0.07, 0.02]	0.11 ± 1.12	−0.10 ± 1.19 ***	−0.21 [−0.08, 0.01]	0.311
BMI-SDS	0.13 ± 1.41	−0.15 ± 1.43 ***	−0.28 [−0.11, 0.03]	0.24 ± 1.54	−0.04 ± 1.62 ***	−0.28 [−0.12, 0.02]	0.322

^1^ Values are Mean ± SD. ^2^ BMI: Body mass index. * Significantly different between the groups by paired-*t* test at * *p* < 0.05, *** *p* < 0.001. ^†^ Group x time interaction effects adjusted for age sex, and type of school (daycare or kindergarten) in the mixed effects linear regression model (random intercept: individual).

**Table 3:** Changes in 19 items of the NQ of the children after participating in the Mission-X program according to their group.

Variables	Intervention Group (*n* = 280)			Control Group (*n* = 254)			
	Baseline	Follow-Up	∆ Change (SD)	Baseline	Follow-Up	∆ Change (SD)	*p* ^†^
Total score ^1^	64.1 ± 10.5	66.0 ± 9.5 ***	2.0 (8.6)	63.7 ± 10.8	63.6 ± 11.6	−0.1 (9.5)	0.050 ^†^
Balance ^2^	56.3 ± 15.6	56.7 ± 14.7	0.4 (11.6)	56.2 ± 14.7	55.9 ± 14.7	1.1 (14.3)	0.907
Wholegrain	57.1 ± 32.2	55.5 ± 31.0	−1.9 (27.9)	57.9 ± 32.6	60.2 ± 32.0	2.7 (24.6)	0.477
Fruit	69.1 ± 22.7	71.1 ± 22.0	2.0 (21.4)	70.9 ± 21.7	69.2 ± 24.0	−1.5 (22.9)	0.050
Milk	64.2 ± 26.1	62.7 ± 28.3	−1.0 (20.1)	59.1 ± 27.5	62.7 ± 27.5 *	3.7 (25.1)	0.272
Legume	42.1 ± 28.7	40.5 ± 24.8	−1.7 (24.1)	41.7 ± 25.2	41.5 ± 23.2	0.5 (25.6)	0.975
Egg	55.1 ± 25.8	54.1 ± 25.3	−1.0 (26.3)	55.7 ± 28.2	57.8 ± 26.0	2.6 (24.1)	0.020
Diversity	60.4 ± 21.8	64.2 ± 21.7 **	3.8 (20.5)	59.2 ± 20.0	58.9 ± 20.5	0.0 (17.1)	0.318
Vegetables	59.4 ± 26.3	61.0 ± 25.5	1.6 (26.6)	59.5 ± 24.3	58.1 ± 24.8	−1.0 (24.5)	0.104
Kimchi	66.5 ± 36.6	71.3 ± 33.5	5.1 (32.4)	67.6 ± 34.7	65.4 ± 35.8	−1.7 (31.1)	0.837
Side dishes	55.7 ± 25.6	61.2 ± 27.8 **	5.5 (27.5)	50.4 ± 27.6	53.3 ± 26.3	3.1 (23.7)	0.758
Moderation	78.2 ± 10.6	78.7 ± 13.6	0.5 (11.9)	77.9 ± 11.9	76.3 ± 12.7 *	−1.2 (11.6)	0.024
Sweet food	41.3 ± 32.5	40.0 ± 32.9	−1.2 (28.7)	37.6 ± 30.2	36.4 ± 27.9	−0.3 (28.8)	0.094
Fast food	82.0 ± 14.2	82.6 ± 17.7	0.8 (18.7)	81.4 ± 14.9	78.8 ± 15.5 *	−1.9 (17.2)	0.325
Instant noodles	85.3 ± 15.1	85.7 ± 19.3	0.3 (18.5)	84.7 ± 14.6	83.5 ± 16.2	−0.9 (13.2)	0.010
Late night snack	88.2 ± 19.8	87.0 ± 24.0	−1.4 (21.0)	90.8 ± 17.7	85.7 ± 23.2 ***	−4.5 (22.1)	0.105
Street food	86.0 ± 18.4	85.0 ± 21.4	−0.6 (21.1)	84.1 ± 20.4	86.7 ± 19.3	2.5 (22.4)	0.795
Regularity	73.7 ± 15.7	74.2 ± 15.9	0.7 (16.6)	74.1 ± 14.8	73.2 ± 15.7	−0.4 (14.8)	0.460
Breakfast	89.5 ± 24.5	90.2 ± 25.3	1.2 (28.5)	91.3 ± 22.7	88.9 ± 25.0	−1.8 (26.6)	0.553
Meal regularity	70.6 ± 18.6	74.0 ± 20.6	3.4 (23.5)	71.5 ± 19.4	72.2 ± 18.7	1.2 (21.2)	0.046
TV watching	45.6 ± 24.9	45.6 ± 22.4	−0.1 (21.5)	46.3 ± 25.2	46.3 ± 25.4	0.5 (18.6)	0.230
Practice	61.8 ± 17.5	65.8 ± 16.6 ***	4.0 (16.3)	60.1 ± 18.1	60.7 ± 17.7	1.1(18.1)	0.162
Chewing	69.6 ± 23.3	73.9 ± 23.4	4.5 (22.5)	67.5 ± 23.8	67.2 ± 23.3	−0.2 (21.7)	0.607
Label reading	41.3 ± 34.5	44.0 ± 31.1	3.0 (30.6)	38.5 ± 35.5	41.5 ± 33.0	4.3 (36.2)	0.808
Washing hands	76.5 ± 23.5	80.7 ± 24.1	4.6 (25.4)	76.2 ± 25.1	75.2 ± 24.5	−0.7 (26.8)	0.000

^1^ Values are mean ± SD. ^2^ Five factors of nutrient quotient (NQ): Balance, diversity, moderation, regularity, and practice. * Significantly different between the groups was found by paired *t*-test at * *p* < 0.05, ** *p* < 0.01, *** *p* < 0.001. ^†^ Group x time interaction effects adjusted for age sex, and type of school (daycare or kindergarten) in the mixed effects linear regression model (random intercept: individual).

## Supplementary Materials

The following are available online at https://www.mdpi.com/2072-6643/11/7/1590/s1, Table S1: Nutrition theme-based Mission X program for South Korean preschoolers.

## References

[1] Birch L.L., Anzman S.L. (2010). Learning to Eat in an Obesogenic Environment: A Developmental Systems Perspective on Childhood Obesity. Child Dev. Perspect..

[2] Hu C., Ye D., Li Y., Huang Y., Li L., Gao Y., Wang S. (2010). Evaluation of a kindergarten-based nutrition education intervention for pre-school children in China. Public Health Nutr..

[3] Larson N., Ward D.S., Neelon S.B., Story M. (2011). What Role Can Child-Care Settings Play in Obesity Prevention? A Review of the Evidence and Call for Research Efforts. J. Am. Diet. Assoc..

[4] Ng M., Fleming T., Robinson M., Thomson B., Graetz N., Margono C., Mullany E.C., Biryukov S., Abbafati C., Abera S.F. (2014). Global, regional, and national prevalence of overweight and obesity in children and adults during 1980–2013: A systematic analysis for the Global Burden of Disease Study. Lancet.

[5] Di Angelantonio E., Bhupathiraju S.N., Wormser D., Gao P., Kaptoge S., De Gonzalez A.B., Cairns B.J., Huxley R., Jackson C.L., Joshy G. (2016). Body-mass index and all-cause mortality: individual-participant-data meta-analysis of 239 prospective studies in four continents. Lancet.

[6] Korea National Health Insurance Corporation (2015). Current Status and Development of Strategic Management of Obesity in Childhood and Adolescence.

[7] Korea Centers for Disease Control and Prevention (2017). 2015 Korea Health Statistics.

[8] OECD (2015). Health at a Glance 2015: OECD Indicators.

[9] Mikkelsen B.E. (2011). Images of foodscapes: Introduction to foodscape studies and their application in the study of healthy eating out-of-home environments. Perspect. Public Health.

[10] Korean Nutrition Society (2015). Dietary Reference Intakes for Koreans.

[11] Kim J.H., Jung Y.H. (2014). Evaluation of Food Behavior and Nutritional Status of Preschool Children in Nowon-gu of Seoul by Using Nutrition Quotient (NQ). Korean J. Community Nutr..

[12] Kim H.K., Kim J.H. (2006). A Preliminary Study on Nutrition Education for Preschool Children in Day-Care Center—Dietary Habit and Nutrition Knowledge. J. Korean Soc. Food Sci. Nutr..

[13] Oh S.M., Yu Y.L., Choi H.I., Kim K.W. (2012). Implementation and Evaluation of Nutrition Education Programs Focusing on Increasing Vegetables, Fruits and Dairy Foods Consumption for Preschool Children. Korean J. Community Nutr..

[14] Choi E.B., Lee J.E., Hwang J.Y. (2018). Fruit and vegetable intakes in relation to behavioral outcomes associated with a nutrition education intervention in preschoolers. Nutr. Res. Pract..

[15] Matwiejczyk L., Mehta K., Scott J., Tonkin E., Coveney J. (2018). Characteristics of Effective Interventions Promoting Healthy Eating for Pre-Schoolers in Childcare Settings: An Umbrella Review. Nutrients.

[16] National Aeronautics and Space Administration (2015). Mission X: Train Like an Astronaut—2014 Annual Report.

[17] Hyunjung L., Jieun K., Youfa W., Jungwon M., Nubia A.C., Charles W.L. (2016). Child health promotion program in South Korea in collaboration with US National Aeronautics and Space Administration. Nutr. Res. Pract..

[18] Kim G.S. (2018). Verification of early childhood health improvement program. Korean J. Sport Sci..

[19] Division of Chronic Disease Surveillance (2018). The Government Report Online 2018 Korean Children and Adolescents Growth Standard (Commentary for the Development of 2018 Growth Chart).

[20] Kang M.H., Lee J.S., Kim H.Y., Kwon S., Choi Y.S., Chung H.R., Kwak T.K., Cho Y.H. (2012). Selecting items of a food behavior checklist for the development of Nutrition Quotient (NQ) for children. Korean J. Nutr..

[21] Kim H.Y., Kwon S., Lee J.S., Choi Y.S., Chung H.R., Kwak T.K., Park J., Kang M.H. (2012). Development of a Nutrition Quotient (NQ) equation modeling for children and the evaluation of its construct validity. Korean J. Nutr..

[22] Wang Y., Cai L., Wu Y., Wilson R.F., Weston C., Fawole O., Bleich S.N., Cheskin L.J., Showell N.N., Lau B.D. (2015). What childhood obesity prevention programmes work? A systematic review and meta-analysis. Obes. Rev..

[23] Kimani-Murage E.W., Kahn K., Pettifor J.M., Tollman S.M., Dunger D.B., Gómez-Olivé X.F., Norris S., Dunger P.D. (2010). The prevalence of stunting, overweight and obesity, and metabolic disease risk in rural South African children. BMC Public Health.

[24] Waters E., De Silva-Sanigorski A., Burford B.J., Brown T., Campbell K.J., Gao Y., Armstrong R., Prosser L., Summerbell C.D. (2011). Interventions for preventing obesity in children. Cochrane Database Syst. Rev..

[25] Wang Y., Wu Y., Wilson R.F., Bleich S., Cheskin L., Weston C., Showell N., Fawole O., Lau B., Segal J. (2013). Childhood Obesity Prevention Programs: Comparative Effectiveness Review and Meta-Analysis.

[26] Adab P., Pallan M.J., Lancashire E.R., Hemming K., Frew E., Barrett T., Bhopal R., E Cade J., Canaway A., Clarke J.L. (2018). Effectiveness of a childhood obesity prevention programme delivered through schools, targeting 6 and 7 year olds: Cluster randomised controlled trial (WAVES study). BMJ.

[27] Commission on Ending Childhood Obesity: Facts and Figures on Childhood Obesity. http://www.who.int/end-childhood-obesity/facts/en/.

[28] Kuh D., Shlomo Y.B. (2004). A Life Course Approach to Chronic Diseases Epidemiology.

[29] Howerton M.W., Bell B.S., Dodd K.W., Berrigan D., Stolzenberg-Solomon R., Nebeling L. (2007). School-based Nutrition Programs Produced a Moderate Increase in Fruit and Vegetable Consumption: Meta and Pooling Analyses from 7 Studies. J. Nutr. Educ. Behav..

[30] Steenbock B., Pischke C.R., Schönbach J., Pöttgen S., Brand T. (2015). The effectiveness of primary prevention interventions promoting physical activity and healthy eating in preschool children. Bundesgesundheitsblatt Gesundheitsforschung Gesundheitsschutz.

[31] Ward S.A., Bélanger M.F., Donovan D., Carrier N. (2016). Relationship between eating behaviors and physical activity of preschoolers and their peers: A systematic review. Int. J. Behav. Nutr. Phys. Act..

[32] Zhou Y.E., Emerson J.S., Levine R.S., Kihlberg C.J., Hull P.C. (2014). Childhood Obesity Prevention Interventions in Childcare Settings: Systematic Review of Randomized and Nonrandomized Controlled Trials. Am. J. Health Promot. AJHP.

[33] Mikkelsen M.V., Husby S., Skov L.R., Perez-Cueto F.J. (2014). A systematic review of types of healthy eating interventions in preschools. Nutr. J..

[34] Esquivel M.K., Nigg C.R., Fialkowski M.K., Braun K.L., Li F., Novotny R. (2016). Influence of Teachers’ Personal Health Behaviors on Operationalizing Obesity Prevention Policy in Head Start Preschools: A Project of the Children’s Healthy Living Program (CHL). J. Nutr. Educ. Behav..

[35] Mouratidou T., Mesana M., Manios Y., Koletzko B., Chinapaw M., De Bourdeaudhuij I., Moreno L. (2012). Assessment tools of energy balance-related behaviours used in European obesity prevention strategies: Review of studies during preschool. Obes. Rev..

[36] Nixon C.A., Moore H.J., Douthwaite W., Gibson E.L., Vögele C., Kreichauf S., Wildgruber A., Manios Y., Summerbell C.D., ToyBox-study group (2012). Identifying effective behavioural models and behaviour change strategies underpinning preschool- and school-based obesity prevention interventions aimed at 4–6-year-olds: A systematic review. Obes. Rev..

[37] Pinket A.S., De Craemer M., Huybrechts I., De Bourdeaudhuij I., Deforche B., Cardon G., Androutsos O., Koletzko B., Moreno L.A., Socha P. (2017). Multibehavioural Interventions with a Focus on Specific Energy Balance-Related Behaviours Can Affect Diet Quality in Preschoolers from Six European Countries: The ToyBox-Study. Nutrients.

[38] Bell L.K., Golley G.R. (2015). Interventions for improving young children’s dietary intake through early childhood setting: A systematic review. Int. J. Child Health Nutr..

