# Supplementary material for: Effectiveness of Teacher-Led Nutritional Lessons in Altering Dietary Habits and Nutritional Status in Preschool Children: Adoption of a NASA Mission X-Based Program
Source: Nutrients. 2019 Jul 13;11(7):1590. doi: 10.3390/nu11071590 (PMC6682966; doi:10.3390/nu11071590)
Supplement: Supplementary file 1 [file nutrients-11-01590-s001.pdf]

Table S1. Nutrition theme-based Mission X program for South Korean preschoolers

week	Nutrition theme	Goals	Activity
1-2	Energy of an astronaut	- Knowing balanced meal based on five food groups<br>- Understanding specific nutritional needs	- Categorizing different food items by five food groups<br>- Pairing food items by five food groups*
3-6	Hydration Station[1)]	- Understanding important roles of water to keep our body healthy<br>- Identifying signs of dehydration<br>- Learning relationship of the body system & organs and hydration	- Think and tell the signs of dehydration and important roles of water in human body<br>- Comparing dried vs. fresh fruits and their peel*<br>- Hydrate the astronaut game*<br>- Observing simulated urine sample*
7	Living Bones, Strong Bones	- Recognizing calcium rich foods	- Comparing different bone shape and structure
8	Reduced gravity, Low-fat	- Formulating healthy food	- Discovering fat & sugar contents
9-10	Class review and practice	- Review the nutritional messages	- Teacher-led class activities based on nutrition theme

[1] Hydration Station: this theme was connected to seasonal events activities (swimming pool or outdoor activities with water) in their ECES

* Based on the nutrition theme, 2, 4-6 and 9-10 week of games and science related classes were delivered by class teachers
